# An experimental study of triggers and needs of threats in critical adversity situations in a student sample

**DOI:** 10.3389/fpsyg.2022.897542

**Published:** 2022-08-30

**Authors:** Mona Rynek, Thomas Ellwart

**Affiliations:** Department of Psychology, University of Trier, Trier, Germany

**Keywords:** threats, stress appraisal, frustrated needs, critical adversity situations, trigger

## Abstract

Emergency teams facing critical adversity situations (CAS) often feel questioned in their professional roles as conscientious rescuers, leading to feelings of threats as a kind of stress experience. According to the stress-as-offence-to-self theory, perceptions of insufficiency and disrespect trigger threats by frustrating underlying needs. In this study, we explored threats in the context of a CAS by investigating the activation of threat triggers during the action and postaction phases of teamwork, and evaluating the mediating role of needs. In a multitask experiment, student teams (*N* = 60 dyads) experienced a controllable mission (non-CAS), followed by a CAS mission in a computer simulation task. After the CAS, teams received negative feedback (situation-nonspecific feedback; situation-specific feedback; no feedback). We measured threats, the activation of insufficiency and disrespect triggers, and the frustration of needs. While insufficiency triggers were activated in the CAS but not in the non-CAS, disrespect triggers were activated by situation-nonspecific and situation-specific feedback but not by no feedback. Furthermore, the results of mediation models indicated the presence of the postulated need-based mechanism between triggers and threats. Our study highlights that the action and postaction phases of a CAS pose a variety of risks for experiencing threats. As individuals cope with these risks, needs are important mediators.

## Introduction

“Police and emergency services are on duty 24/7 to protect each and every one of us. Yet they are often hindered in their work, insulted, or attacked” (Federal Ministry of the Interior, Building and Community, Germany, 2017). Stressful work events often cause members of emergency teams performing in critical adversity situations (CAS) to feel threatened in their role as reliable and conscientious rescuers (e.g., Feltes and Weigert, 2018; BBC News, 2019). As a consequence, the emergency workers become exhausted, even though they are doing the most important jobs for society (e.g., Anshel, 2000). Improving the understanding of threats in CAS and identifying approaches to assist emergency teams in coping with the triggers and mechanisms of threats are topics of interest.

Threats are a kind of stress experience (Tuckey et al., 2015; Semmer et al., 2019). According to the stress-as-offence-to-self theory (SOS, Semmer et al., 2007, 2019), threats occur because of perceptions of insufficiency (i.e., the experience of failure) and disrespect (i.e., devaluation by others) that frustrate underlying needs (e.g., the need for relatedness). Mainly, interview and survey studies (e.g., Feltes and Weigert, 2018) imply that especially emergency teams are exposed to these triggers of threat. Although emergency teams receive intensive training, the specific characteristics of CAS such as complexity and dynamic (Semling and Ellwart, 2016) cause them to fail or behave inappropriately (e.g., inaccurate shooting, Anshel, 2000), or experience disrespect by verbal attacks of the public (Anshel, 2000). However, not only in the CAS itself (action phase) but also in phases of team reflection and feedback (postaction phase), the experience of insufficiency and disrespect is not unlikely, for instance by reprimands of the supervisor (Anshel, 2000). Since triggers potentially occur in different phases of their teamwork, i.e., during and postaction (*cf.* Marks et al., 2001), the danger of threats posed to emergency teams seems to be manifold. To explain why individuals feel threatened by insufficiency and disrespect as triggers, Semmer et al. (2007) refer to an offended general need for positive self-views but also specific frustrated needs for competence and relatedness. Numerous motive approaches postulate further needs such as the need for control (*cf.*, McClelland, 1961; Deci and Ryan, 2000), which might affect threat experiences of teams working in CAS context (Regehr and Millar, 2007).

In this study, we consider different manifestations of insufficiency and disrespect as threat triggers in the context of CAS. Furthermore, we consider triggers from a temporal perspective (Marks et al., 2001) and examine whether situational characteristics of the action phases (CAS vs. non-CAS) and different kinds of feedback styles used during postaction phases (situation-specific vs. situation-nonspecific vs. no feedback) activate insufficiency and disrespect as triggers of threats in an experimental setting. Furthermore, we operationalize basic human needs that might be particularly relevant in CAS and investigate them as underlying mediators between triggers and threats.

This study extends the research in several ways. First, by considering triggers postulated in the SOS theory (Semmer et al., 2019) in the specific context of CAS, we highlight that there is a great potential of threats not only during the CAS itself but also afterward. Second, we experimentally confirm the need-based threat mechanism assumed by the SOS theory and emphasize the importance of triggers and needs for the experience of threats. Through the experimental design of our study, causal interpretation of the trigger-need-threat mediation is possible. Third, at a practical level, our study highlights that the action and postaction phases of CAS pose a variety of risks for experiencing threats within and after an emergency scenario. To help emergency teams cope with these risks, specific needs are important mediators. The frustration of specific needs explains why threat experience occurs. Consequently, reattribution and reflection is an approach used to reduce need frustration and the occurrence of threats in the case of activated triggers in the context of a CAS.

## Threat mechanism: Triggers and need frustration

**Figure 1:**
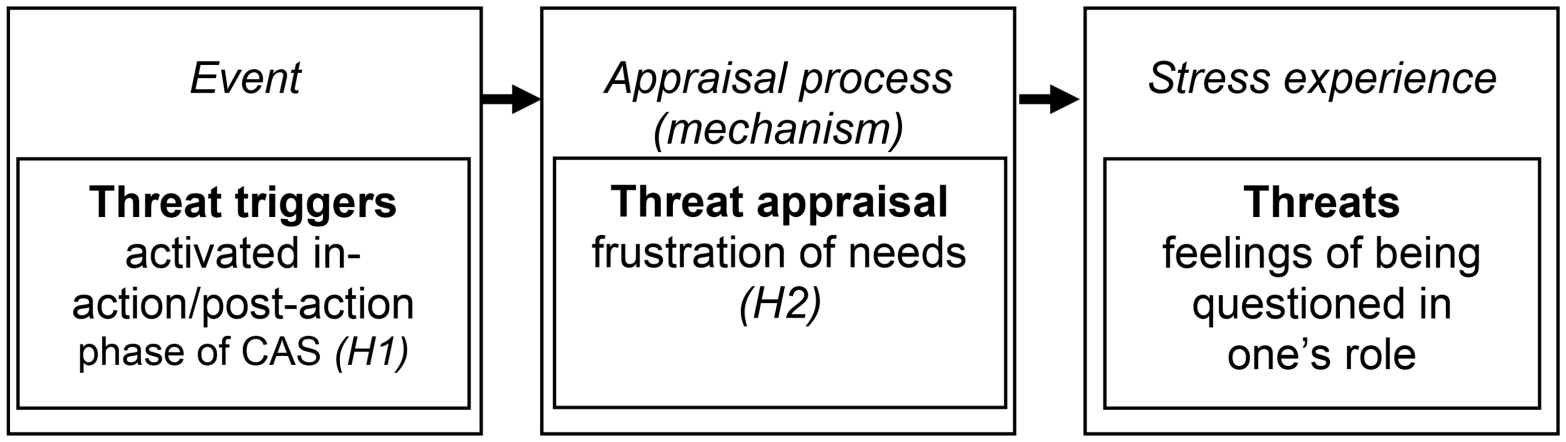
Overview of determinants in the threat process.

Although threats are most commonly thought of as a feeling triggered by a physical attack, various research disciplines describe threats as a kind of stress experience (Tuckey et al., 2015; Semmer et al., 2019). Individuals can appraise stressful events as challenges (i.e., a feeling of mastery or gain), as hindrances (i.e., feeling hindered in goal attainment), or as threats (i.e., feeling harmed or questioned in one’s role); all are experienced as a kind of stress. Therefore, emergency workers who experience threats concerning stressful events may do so because feel questioned or harmed in their professional role as reliable and conscientious rescuers (Figure 1). According to SOS theory, this type of stress experience can be explained by (a) triggers and (b) frustrated needs as a mechanism.

According to the SOS theory (Semmer et al., 2007, 2019), insufficiency and disrespect are two triggers of threats. The perception of disrespect originates from events in which others’ behaviors signal a lack of respect and appreciation. There is a wide range of disrespectful behaviors, including rude feedback, making someone lose credibility in the presence of others, ignoring someone, withholding social support, or in a more indirect way assigning unnecessary tasks or providing inadequate technology (Semmer et al., 2007). The perception of insufficiency results from events in which individuals experience failure and internally attribute this failure to a lack of abilities and skills (Semmer et al., 2007, 2019). Insufficiency often manifests in situations demanding high performance (such as events in which others evaluate the individual’s abilities and skills negatively in terms of attaining performance-related goals, causing them to perceive themselves as insufficient) or situations demanding ethical behavior (such as events in which individuals evaluate their skills and abilities in terms of moral appropriateness in the given situation, Semmer et al., 2019). Therefore, a variety of events are perceived to potentially provoke feelings of insufficiency or disrespect (i.e., activate insufficiency and disrespect as threat triggers).

Individuals experience threats in the face of these triggers due to appraisal processes (e.g., Tuckey et al., 2015), which means that individuals appraise stressful events (triggers) as threatening (Figure 1). While a variety of factors (e.g., attachment, beliefs, values) are discussed as influencing emotional or motivational appraisal processes in general (Ellsworth and Scherer, 2003), the frustration of needs (e.g., needs for competence, relatedness, or autonomy) is specifically cited as an important determinant concerning the appraisal of threats (Ntoumanis et al., 2009). Needs are more than goals that individuals try to reach (e.g., “as a paramedic, I want to be helpful,” Deci and Ryan, 2000). Rather, needs are internal forces that are essential for supporting life and growth. If they are unmet, they create tension and stimulate drives within the individual (Kanfer et al., 2017); they also have negative cognitive, emotional, and motivational effects (e.g., Olafsen et al., 2017; Manganelli et al., 2018). For example, Quested et al. (2011) show that frustrated needs reported as threats were accompanied by feelings of anxiety. Additionally, in the framework of the SOS theory (Semmer et al., 2007, 2019), threats are explained by an underlying mechanism of need frustration.

While Semmer et al. (2019) refer to a global need for a positive self-view, Semmer et al. (2007) include more specific needs, such as the needs for competence and relatedness, to explain threats. The need for competence describes an individual striving to be effective in the achievement of desired outcomes (e.g., to have moral strength, to be competent, to fulfil ideal self-representations), which is frustrated by perceptions of insufficiency (Semmer et al., 2007). Individuals perceive that they do not meet their standards or ideals of self-representation. As a result, they cannot think of themselves as capable and effective individuals; instead, they experience feelings of failure and doubts about their efficacy, so their need for competence is frustrated. The need for relatedness describes an individual striving to belong to a given group (i.e., to have close relationships with others), which is frustrated by perceptions of disrespect (Semmer et al., 2007). By facing disrespect, individuals perceive themselves as being treated poorly, ignored, or excluded instead of experiencing intimacy and genuine connection to others, which frustrates their need for relatedness. Semmer et al. (2019) suggest that the content of motives and needs should be further investigated, based on the fact that numerous motivational approaches (e.g., Self-determination theory, Deci and Ryan, 2000; Motive-Disposition Theory, McClelland, 1961) postulate further strivings of an individual (e.g., need for autonomy, Deci and Ryan, 2000).

So far, there is substantial evidence that generally confirms the postulates of the SOS theory (Semmer et al., 2019). Investigations of how triggers manifest in a specific context such as CAS, which specific needs are particularly relevant in dealing with CAS, or the consideration of the relationship of multiple triggers and needs, have not yet received attention in research, up to our knowledge. However, in order to be able to support emergency teams appropriately in dealing with threats or to be able to avoid threats, it seems reasonable to analyze the work context more precisely in terms of triggers and relevant needs. The CAS context reflects the everyday work of emergency teams such as police officers, firefighters, paramedics, etc., and has special characteristics: First, the CAS context is characterized by the alternation of action phases describing the active task execution in the emergency situation itself, and less active postaction phases, in which team reflection and feedback are the focus (Marks et al., 2001). Second, CAS-related action phases are characterized by specific situational characteristics such as dynamic, complexity, and ambiguity (Semling and Ellwart, 2016). Third, dealing with CAS means always to interact with individuals or groups, such as other emergency teams, victims and perpetrators, colleagues and superiors, and the media (Anshel, 2000) doing this in action as well as postaction phases.

Reasons why emergency teams experience disrespect and insufficiency in these specific CAS contexts, how both triggers manifest in the context of CAS, and the frustration of which needs may be particularly important in CAS, are described below.

## Triggers of threat in CAS

The emergency team’s frequent experience of insufficiency can be explained by considering the situational characteristics of the CAS. A CAS is characterized by complexity, dynamism, time pressure, and poses undefined and complex demands for which there is no perfect solution (i.e., ill-defined problems, Wildman et al., 2012). Therefore, the question of what represents the appropriate action in a CAS cannot be answered unambiguously, so the evaluation of being successful or failing remains open as well (Semling and Ellwart, 2016). Moreover, teams working under CAS circumstances have high responsibility (e.g., Hagemann et al., 2012), as being insufficient in one’s role has serious consequences for themselves and others (e.g., Maynard et al., 2018). This even makes them liable for mistakes (Marsden et al., 2020). As a result, emergency teams strive to master the CAS successfully. However, despite their best efforts, they also exhibit erratic behavior in the CAS, such as inaccurate shooting (Anshel, 2000), or they perceive that their skills are not sufficient, as reported by the workers referring to a lack of experience with the computer in the rescue vehicle (Jacobsson et al., 2015). They might perceive themselves as insufficient due to a failure in the CAS itself (during the action) based on their own skills and abilities, even if the situational characteristics of the CAS have influenced the wrongdoing.

According to Petriglieri (2011), situational characteristics can also trigger threats without being attributed as an internal failure. However, the fact that the situational characteristics of a CAS (e.g., complexity, ambiguity; Semling and Ellwart, 2016) appear threatening can also be explained by insufficiency perceptions. Based on the threat-rigidity thesis (Staw et al., 1981), individuals respond to stressful demands, such as those a CAS places on individuals, with impaired information processing. Thus, instead of having different environmental factors in mind and adapting as would be required to successfully master a CAS (Maynard et al., 2018), the characteristics of the CAS lead to a narrowed field of attention, a reduction in the number of alternatives considered, and further actions that make failure likely (Kamphuis et al., 2011). Thus, hindrances in action experienced due to the situational characteristics of CAS might also lead to feelings of insufficiency and thus to threats while handling the CAS (i.e., action). In this respect, we identify different forms of insufficiency perceptions in the CAS context: the experience of failure (i.e., internal wrongdoing due to skills and abilities) and hindered-action regulation (i.e., external wrongdoing due to contextual and environmental factors).

That emergency teams experience disrespect can be explained by referring to the fact that they interact with different people and groups, both inaction and postaction phases. While handling a CAS, disrespect manifests usually by (verbal) attacks from the public. For example, Jacobsson et al. (2015) reported that firefighters feel disrespected in their duty by being assaulted and exposed to violence such as stone-throwing and public verbal attacks. According to respect research (Grover, 2013, 2021; Decker and Van Quaquebeke, 2015), this lack of receiving unconditionally guaranteed and dignified treatment is one of two dimensions of disrespect, which we term relationship-related disrespect. Relationship-related disrespect may manifest primarily in the CAS itself (during action) as the workers are dealing with the public, perpetrators, victims, or bystanders (Duran et al., 2018).

Furthermore, there is evidence that emergency teams experience disrespect in postaction phases of a CAS *via* feedback or a lack of support from supervisors (Anshel, 2000; Jacobsson et al., 2015). Krings et al. (2015) show that feedback need not even include offensive statements made in an inconsiderate tone to be perceived as disrespectful (as is the case in behavior that is categorized as relationship-related disrespect). The authors report that dwelling on or exaggerating mistakes or suggesting that nothing was easier than avoiding a given mistake can also be perceived as disrespectful, even if it is expressed in an appropriate tone. Here, disrespect is not related to human dignity but rather to the valuation of an individual’s excellence or expertise and thus relates to the performance and effort an individual has contributed. According to respect research, this kind of disrespectful behavior describes another dimension of respect (Grover, 2013, 2021; Decker and Van Quaquebeke, 2015), which we term performance-related disrespect. Performance-related disrespect manifests in interactions between team members or supervisors “because making judgments about others is essential to performance management and leadership” (p. 35, Grover, 2013). Therefore, the danger of performance-related disrespect also may exist in the reflection and feedback phases after a CAS (postaction).

To our knowledge, no study examines the different manifestations of insufficiency and disrespect triggers in the context of CAS and their appearance in different teamwork phases (action and postaction, Marks et al., 2001) to understand in more detail where the danger for threat exists for teams working in CAS.

## Frustrated needs in CAS

Police officers, firefighters, paramedics, etc., have an important job for society and classify the responsibility entailed as demand that requires effort (e.g., Gudjonsson, 1984; Nedzinskas et al., 2020). In line with the Effort-Reward-Imbalance Model (Siegrist, 1996), they expect to be appreciated and valued by others for this effort. Thus, emergency workers might not only seek to belong to others but also receive some recognition and appreciation. Respect research describes this individual striving as a need for status, which is distinguished from the need for relatedness. In the dual pathway model of respect, Huo et al. (2010) differentiate the need for relatedness and the need for status by describing two pathways through which respect shapes attitudes and behavior. Whereas the need for status describes the degree to which others value and appreciate an individual, resulting in social engagement, the need for relatedness describes the degree to which others like an individual, resulting in personal well-being. The need for status might be important in the context of teams working in CAS in addition to the need for relatedness.

Furthermore, due to the specific characteristics of the situation, a striving for control might also be important in the context of CAS. Regehr and Millar (2007), for example, point out that paramedics’ ability to control the situation is important to label a situation as successfully mastered. The desire to have a way of determining what and how something is done and how resources are allocated and used is described, for example, in self-determination theory (Deci and Ryan, 2000) by the need for autonomy or in motive-disposition theory by the need for power (Schüler et al., 2019). Because the concept of control is appropriate in the context of situational characteristics such as CAS, we refer to this individual striving as the need for control. Quested et al. (2011) already associate such a desire for control with threats. In addition to the need for competence, the need for control might be important for threat experiences in the context of CAS.

In addition to the needs for competence and relatedness mentioned in the SOS theory, status and control needs might also be frustrated for teams working in CAS. The extent to which these needs are frustrated in CAS and are even responsible for the threat experience has not yet been investigated.

## The current study

In this study, we explore (1) different forms of insufficiency and disrespect triggers in the context of CAS and look more closely at where the danger for threat exists among teams working in CAS, (2) we examine specific needs that may be relevant to experiencing threat in the context of CAS to find approaches for avoiding and dealing with threats. For this, we assume the following:

As illustrated, we have two types of insufficiency, the experience of failure and hindered action regulation, which might be relevant for threat experiences in the CAS context. In more detail, both types of insufficiency appear to be directly related to action phases of CAS, and thus, both more or less directly depend on situation characteristics. In our experimental study, we compare perceptions of insufficiency in CAS and controllable situations (non-CAS) and take into account that perceptions of insufficiency can be activated internally as an experience of failure and externally as hindered action regulation. We hypothesize the following:

*Hypothesis 1a*: In an uncontrollable CAS but not in a non-CAS, hindered action regulation and experiences of failure are activated as triggers of insufficiency.

Also in relation to disrespect we have two types, which might be relevant for threat experiences in CAS context: relationship-related disrespect and performance-related disrespect. Relationship-related disrespect might be mainly associated with the action phase, where people are verbally attacked by the public. But especially the second type, performance-related disrespect, might also occur in postaction phases. Thus, in this study, we focus on the investigation of disrespect experiences in postaction phases and aim to show that feedback in postaction phases of a CAS can activate disrespect triggers. However, the question arises: what makes feedback after a CAS appear disrespectful concerning performance in the CAS?

According to Semmer et al. (2016, 2019), disrespect is experienced when the feedback recipient feels his or her interests are neglected and the feedback provider expresses disinterest (Semmer et al., 2016, 2019). In the context of a CAS, not respecting the stressful situation characteristics of the CAS in the feedback might lead to the attribution of such disinterest and, thus, to disrespect perceptions on the feedback recipient’s side. Conversely, putting oneself in the situation of other people (i.e., those involved in the CAS) and evaluating their performance considering the stressful situation characteristics of the CAS (e.g., uncontrollable, complex, dynamic) might signal interest in the counterpart’s experience and feelings, which is perceived as respectful. Van Thielen et al. (2018), who investigate teams in CAS, support this assumption. The authors show that negative feedback is constructive and helpful when the feedback provider is tactful, supportive, and considerate of the feedback receivers’ feelings.

The feedback provider’s inclusion of the situation specificity of the CAS in the feedback might be the key determinant for the perception of feedback after a CAS as respectful. Conversely, neglecting the situation specificity of CAS in feedback after a CAS might be the key determinant that activates perceptions of disrespect. In this experimental study, we examine whether feedback after a CAS activates disrespect as a threat trigger. In more detail, we investigate situation specificity as a crucial determinant of perceiving feedback after a CAS as disrespectful, and we consider the idea of respect research by taking different kinds of disrespect (i.e., performance-and relationship-related disrespect) into account. Since negative feedback, in particular, is associated with stress and threat experiences (e.g., Burnette et al., 2010; Dunn and Dahl, 2012; Krings et al., 2015; Lee-Won et al., 2017), we examine the effects of negative situation-specific feedback, compared to negative situation-nonspecific feedback and no feedback, on trigger activation.

*Hypothesis 1b*: In the postaction phase of a CAS, negative situation-nonspecific feedback activates performance-and relationship-related disrespect triggers compared to negative situation-specific feedback and no feedback.

By examining frustrated needs as a mechanism, we assume the following specific combinations of triggers and needs:

Insufficiency triggers might be related to the need for competence. Individuals strive to explain their own and other individuals’ behavior and engage in attribution processes. They attribute causes to the experience of insufficiency (Weiner, 2014). If individuals internally attribute insufficiency to their own behavior, identifying their aptitude or talent as causes for the insufficiency, this directly concerns their appraisal of competence (Perry and Hamm, 2017). For example, if a police officer handling a CAS attributes an inaccurate shooting to his or her lack of abilities in shooting, this is accompanied by feelings of incompetence. Thus, a frustrated need for competence may be the result of an internal attribution in insufficiency.

Furthermore, insufficiency triggers might be related to the need for autonomy. If individuals perceive their actions as independent of the outcome (i.e., if they feel that they cannot determine the outcome with their actions), this leads to the perception of helplessness, a kind of loss of control (*cf.* Seligman, 1975). The general feeling of being hindered in action is also associated with feelings of loss of control, which is an unpleasant state that individuals try to avoid (*cf.* Brehm, 1966). For example, if a police officer handling a CAS attributes an inaccurate shooting to being hindered in action (e.g., “I stand with my back to the wall…”), this is accompanied by feelings of a loss of control. Thus, a frustrated need for autonomy might also be the result of perceiving insufficiency.

Integrating this relationship between perceptions of insufficiency and the need for competence and autonomy into the trigger-need mechanism, insufficiency perceptions might frustrate not only the need for competence but also the need for autonomy, resulting in threats. We postulate:

*Hypothesis 2a*: The need for competence and the need for autonomy mediate the relationship of insufficiency triggers (experiences of personal failure and hindered action regulation) on threats after facing a CAS.

Disrespect triggers might be related to the needs for status and relatedness. Respect research indicates specific connections between (dis)respect and the needs for relatedness and status (Huo et al., 2010). In their dual pathway model of respect, Huo et al. (2010) assert that respect is mediated *via* the need for status (i.e., the desire for being valued by others) or the need for relatedness (i.e., the desire for being liked by others) and that it shapes attitudes and behaviors. If individuals are not treated fairly, they perceive that they are not worthy and valued members of a group, implying a low status. Similarly, individuals who are treated unfairly may also perceive that they are not liked by others, implying a feeling of low relatedness. Blincoe and Harris (2011) confirm the relationship between a lack of respect and frustrated needs for status and relatedness.

Integrating this relationship between perceptions of disrespect and the need for relatedness and status into the trigger-need mechanism of the SOS theory (Semmer et al., 2007), disrespect perceptions might frustrate not only the need for relatedness but also the need for status, which, in turn, lead to threats. We postulate:

*Hypothesis 2b*: The need for relatedness and the need for status mediate the relationship of disrespect triggers (relationship-and performance-related disrespect) and threats after facing a CAS.

## Materials and methods

### Participants

Participants included *N* = 120 students from a German university. Students from different disciplines were recruited *via* advertisements on campus and the university student participant recruitment system. All participants voluntarily attended this experiment, which was approved by the Ethical Committee of the German university. Participants were informed that this was a study in which they had to master a firefighting simulation game on a PC as a part of a team. In the sample, 73.33% of the participants were female. Their mean age was 21.66 years (*SD* = 2.69). Students came from different disciplines and had no experience working in emergency services. All students participated in teams of two in the experiment and received course credit. They were able to register for the study in pairs, so in 73.33% of the teams, the team members were friends, in 8.33% of the teams, they knew each other briefly, in 16.73% of the teams, they did not know their team partner, and 1.73% of the team members did not provide any information on their relationship. About 95% of the participants had no experience with the computer simulation task used to create the experimental setting. About 5% of the participants had already used the simulation task once in a previous study.

### Task: Characteristics of CAS

To implement a CAS in the experimental setting, we used the Networked-Fire-Chief computer simulation (NFC32 V1.42; Omodei et al., 1996) and stimulate an interdependent team-task-situation. Originally, the software was developed to examine psychological processes in complex, dynamic, and uncontrollable situations. The simulation runs to two networked computers simultaneously so that team members work together from their individual computers. The teams’ task was to fight emerging fires on a map of a village environment. Team members had three fire trucks and three helicopters at their disposal for extinguishing fires. After use, they had to be refilled with water by positioning them at the lake. Situation characteristics (CAS vs. Non-CAS) are varied through the number of fires and the speed at which they spread (e.g., CAS was implemented by a high number of fast-spreading fires). The functioning of the simulation can be learned easily. The computer simulation task has been used previously in several studies where different levels of complexity/controllability were tested (e.g., Timm et al., 2020). Thus, we were able to select uncontrollable (Non-CAS) and controllable simulations (CAS).

### Procedure

**Figure 2:**
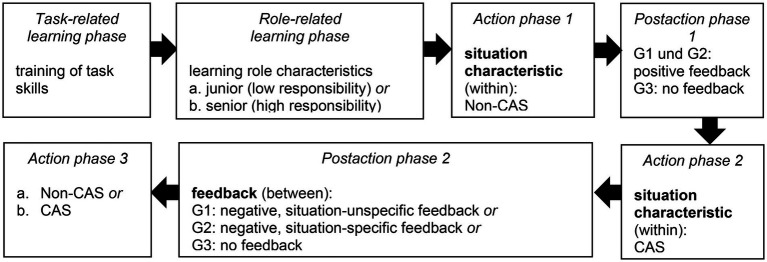
The experimental procedure.

Participants came to the laboratory. The experimenter informed them that the study examines the communication and coordination processes of emergency teams, such as firefighters, paramedics, and police, and the participants have to take on the role of a firefighting team and extinguish fires. Then, they were informed about the procedure. The study included a task-and role-related learning phase, three action phases, and two postaction phases (see Figure 2) and lasted ~90 min for each team. The first action phase served as a baseline in which the subjects were confronted with a situation that was easy to handle so that they were able to act based on what they had learned in the learning phase. Action phase 2 allowed the manipulation of a CAS. The subsequent action phase 3 was another experimental control phase.

Participants sat opposite each other at two computers in one room. They started with a task-related learning phase (similar to Uitdewilligen et al., 2013), in which they were introduced to the use of the Networked-fire-chief-simulation. After a standardized presentation, there was an 8-min practice trial, during which the participants could familiarize themselves with the functioning of the simulation. The participants then evaluated their task skills in a questionnaire. In the subsequent role-related learning phase, first, the participants were presented with images from firefighting operations (e.g., firefighters unroll their hoses, firefighters splash water on a burning house) to better put themselves in the firefighting setting. Then, they received information about four key characteristics of the firefighter role (e.g., to be helpful) *via* a presentation. Roles were randomly assigned. To ensure a uniform, realistic, and structured task execution, we assigned responsibility as an additional key role characteristic to one participant as a senior firefighter (they are responsible – high responsibility) and one participant as a junior firefighter (the team partner is responsible – low responsibility). The senior team member received extra information (e.g., to monitor the environment) and was instructed to make decisions for the team in the case of a CAS. This role-related learning phase was followed by a test of their knowledge of their role’s characteristics, behavior, and identification to check competencies and commitment in the role of a firefighter.

After the task-and role-related learning phases, which were the same for all teams, participants started their team tasks. They were allowed to talk to each other and were instructed to fight emerging fires together. In the three action phases, which were separated by two postaction phases, every team passed through the Networked-fire-chief-simulations with two different situation characteristics, i.e., non-CAS and CAS (independent variable 1). In action phase 1, all teams faced a non-CAS operationalized by a few slow-spreading fires. This non-CAS was easy to handle so that teams had a successful first mission. During the following postaction phase 1, two-thirds of the teams received positive team feedback from the principal investigator (e.g., “You managed the situation successfully.”). One-third of the teams in the control group (G3) received no feedback. With regard to H2a, in the subsequent action phase 2, a CAS was operationalized by many fast-spreading fires. This CAS led to overburdening, decision dilemmas, and failures of the team members’ role requirements (i.e., team members were not able to behave according to their role characteristics, for instance, being too slow to extinguish all fires). Concerning H2b, in the following postaction phase 2, the G3 again did not receive any feedback. For the other teams, the experimenter provided different styles of feedback (independent variable 2) so that teams received either negative, situation-nonspecific feedback (G1) or negative, situation-specific feedback (G2). In G1, the experimenter evaluated the team’s performance negatively and ignored situational characteristics (e.g., by simply saying: “You failed.”). In the negative, situation-specific feedback (G2), experimenter gave feedback consisting of a negative evaluation (same in G1) that also referred to situational characteristics (e.g., “The situation was difficult to handle.”). After transition phase 2, half of the teams experienced another CAS, and half of the teams experienced a non-CAS. This action phase 3 was implemented to show that there was no learning curve, because we did not randomize the sequence of non-CAS- and CAS-situation characteristics across action phases 1 to 3.

The participants completed a questionnaire during transition phases 1 and 2. Immediately after the participants finished the simulation in action phases 1 and 2, we checked our manipulation of the situation characteristics. After the teams received feedback, we checked our manipulation of the feedback and measured the threat, beliefs and concerns related to the handling of the current CAS and a future CAS, the activation of triggers, and the satisfaction of needs.

### Measures

Participants rated all responses on a six-point Likert scale, ranging from 1 (strongly disagree) to 6 (strongly agree). Since we derived scales used for measuring threat, trigger activation, and needs in the context of CAS from theoretical definitions (*cf.* Rynek and Ellwart, 2020) or adapted along with validated scales (e.g., Basic psychological need satisfaction scale—BPNSS, Deci and Ryan, 2000), validity was ensured using expert interviews and correlates. All items used in the questionnaire can be requested from the corresponding author.

*Threat*. Four items assess the participants’ perception of the extent to which they felt questioned in their role as a firefighter (e.g., “I feel questioned in my role.”; α = 0.79).

*Trigger activation*. To capture experience of failure as a perception of performing inadequately, we used three items (e.g., “I was not able to achieve the desired results”; α = 0.82). We used three items to measure hindered action regulation. Participants rated the extent to which they felt hindered in carrying out their actions (e.g., “I did not know how to act.”; α = 0.76). For measuring performance-related disrespect, we used three items indicating a lack of respect that refers to an individuals’ expertise and performance (e.g., “I got the recognition I deserve for my performance.”; α = 0.74). Relationship-related disrespect was measured by three items. Participants rated the extent to which they felt equally treated between individuals (e.g., “Others conveyed to me with their behavior that they did not think anything of me [as a person].”; α = 0.87).

*Needs*. Items measuring needs satisfaction were based on validated scales (e.g., BPNSS, Deci and Ryan, 2000) and express the participants’ satisfaction with regard to the fulfillment of their needs. Three items measured the need for competence by referring to participants’ desire for the craving to accomplish desired outcomes and for being able to act effectively (e.g., “I had the feeling that I was competent.”; α = 0.89). Three items measured the need for autonomy as the participants’ desire to be the perceived origin of one’s behavior (e.g., “I had the feeling that I was in control of what I was doing.”; adapted from the need for autonomy of BPNSS; α = 0.78). The need for relatedness was measured by three items that indicate the participants’ desire for connectedness to other people (e.g., “I had the feeling that I belonged to others.”; α = 0.83). We used three items measuring the need for status referring to the participants’ desire for a positive standing and worth in a group (e.g., “I had the feeling that I was an important person for others.”; α = 0.86).

*Manipulation checks*. For checking the manipulation of situation characteristics, participants rated their perception of the situation based on five characteristics (complexity, dynamic, time pressure, resource availability). The manipulation of feedback was checked with five items (e.g., “The principal investigator judged me negatively without considering the situation”), similar to Krings et al. (2015), who also asked their participants to evaluate the feedback.

We checked task skills at the end of the task-related learning phase. Both knowledge and skills to handle the simulation were evaluated by two items (“I know how fire extinguishing works”; “If a fire emerges in the simulation, I can extinguish it”). Furthermore, we checked several aspects related to role-learning: role knowledge, role identification, and role behavior. Before starting action phase 1, we checked the participants’ role knowledge. Participants had to list their role characteristics and answered the question of who makes the decisions in CAS. Participants had to rate the extent of role identification (“How well do you identify with the role as a firefighter/as a senior being responsible?”). Furthermore, we checked whether the participants would apply their role characteristics in an emergency situation (role behavior). To do this, the participants imagine an emergency situation, in which they act as firefighters and judge their behavior in this situation. For this, their role characteristics were presented as dimensions (e.g., helpful … no helpful, fast … slow). On a Likert scale with values between 1 and 5, values of 5 indicated role-consistent behavior, while values of 1 indicated role-inconsistent behavior. After action phases 1 and 2, participants judge their behavior in the action phases also along with these dimensionally presented role characteristics to check whether they have behaved according to their role characteristics.

### Statistical analyses

To check the situation characteristic and feedback manipulation, we calculated group differences using *t-tests* in IBM SPSS Statistics 26 (Version 26.0.0.1). To test hypothesis 1a, we conducted a MANOVA with repeated-measures comparing trigger activation in non-CAS and CAS. This MANOVA was calculated only for the no-feedback group (G3) to avoid confounding effects with feedback manipulation. A second MANOVA comparing trigger activation of the three feedback groups (G1, G2, and G3) provides results for hypothesis 1b.

Before testing hypotheses 2a and 2b, we analyzed the internal structure of triggers and needs to show that they represent independent constructs. We conducted a confirmatory factor analysis (CFA) with a lavaan package in RStudio (Version 1.2.5019) and compared the properties of two structural models: Model 1 represents a g-factor model, where all items load on a single general factor (g-factor). Model 2 represents a first-order correlated factor model and contains two different but correlated factors that correspond to triggers and needs. We hypothesized that Model 2 fits the data better than Model 1. In both CFAs, we specified five triggers and five needs and the g-factor or a general trigger-and a general need-factor as latent variables. To handle convergence problems, the unstandardized loading of the first item of each first-order factor was fixed to 1. We then evaluated both structural models based on different indices. We followed Kline (2015), Hu and Bentler (1999), and Dimitrov (2010) by evaluating the chi-square goodness-of-fit, comparative fit index (CFI), Tucker–Lewis index (TLI)—the values for both models should be above 0.95, the root-mean-square error of approximation (RMSEA), the standardized root mean squared residual (SRMR)—the values for both Model 1 and Model 2 should be below 0.08 and 0.06, respectively, Akaike information criterion (AIC), and Bayesian information criterion (BIC). We did not have any missing data on any of the scales. The models were nested, so model fit could be compared by using the chi^2^-differences test.

To test hypotheses 2a and 2b, we, first, conducted parallel mediation models for each trigger as independent variables, the four needs as parallel mediators, and threat as the dependent variable. We conducted nonparametric bootstrapping analyses using PROCESS macro by Preacher and Hayes (2004, 2008); Model 4, Hayes, (2013), which uses ordinary least squares regression, yielding unstandardized path coefficients for total, direct, and indirect effects. Bootstrapping analyses are suited for smaller samples and do not impose the assumption of normality on the sampling distributions (Hayes, 2013). In detail, within the bootstrap test, we repeated the estimation of our structural models 5.000 times. The mediator effect is significant if the 95% bias-corrected and accelerated confidence interval (BCa CI) for the indirect effect does not include zero (Hayes, 2013). Due to the correlations between the four needs (results from bivariate correlations in Table 1), we found no mediation effects for the parallel mediation models. Thus, we conducted separate mediations for each trigger-needs (independent variable-mediator)-combination, with threat as an outcome.

**Table 1:** Descriptive statistics and correlations of threat, triggers, and needs after CAS (action phase 2).

Scale	*M*	*SD*	Min	Max	1	2	3	4	5	6	7	8	9
1. TH	2.28	0.98	1.00	5.25	(0.79)								
2. EOF	4.59	0.91	1.67	6.00	0.27*	(0.82)							
3. HAR	3.34	1.22	1.00	6.00	0.26*	0.29*	(0.76)						
4. PRD	2.33	0.92	1.00	6.00	0.69*	0.11	0.24*	(0.74)					
5. RRD	1.13	0.36	1.00	3.00	0.43*	0.12	0.38*	0.49*	(0.87)				
6. COM	2.87	0.85	1.00	6.00	−0.31*	−0.61*	−0.41*	−0.12	−0.11	(0.89)			
7. AUT	3.41	1.01	1.00	5.67	−0.31*	−0.32*	−0.42*	−0.13	−0.13	0.56*	(0.78)		
8. STA	4.47	0.99	1.33	6.00	−0.39*	−0.18*	−0.34*	−0.32*	−0.28*	0.40*	0.53*	(0.86)	
9. REL	5.33	0.67	2.00	6.00	−0.29*	−0.04	0.15	−0.27*	−0.22*	0.17	0.23*	0.56*	(0.83)

TH = threat, EOF = experience of failure, HAR = hindered action regulation, PRD = performance-related disrespect, RRD = relationship-related disrespect, COM = need for competence, AUT = need for autonomy, STA = need for status, REL = need for relatedness; M = mean, SD = standard deviation, Min = Minimum, Max = Maximum. ^*^ indicates *p* < 0.05. Interne consistence (Cronbach’s alpha) in parenthesis.

## Results

### Preliminary results

#### Checking the learning procedure: Task skills

A total of 97.48% of the participants stated that they knew how firefighting works (answered with rather agree or agree), and 95.83% of the participants also attempted to extinguish a fire as soon as it appeared on the screen (answered with rather agree or agree). The performance changed little over action phases 1 to 3. In action phase 1, subjects saved an average of 99.69% of the surface by extinguishing the fire during the non-CAS. By comparison, in action phase 3, they saved 99.88% of the surface during the non-CAS. Statistically, we found a significant difference between the performance in a non-CAS in action phase 1 and action phase 2, *t*(59) = 13.07, *p* < 0.001. In the CAS in action phase 2, the subjects saved an average of 83.51% of the surface by extinguishing fires. When the subjects in action phase 3 were confronted with a CAS, they saved 86.98% of the surface. Statistically, we found a significant difference between the performance in a CAS in action phase 2 and action phase 3, *t*(59) = −2.46, *p* < 0.017.

#### Checking the learning procedure: Role

A total of 72.50% of the participants correctly named at least half of the role characteristics, and 97.50% of the participants correctly identified the senior firefighter as the decision-maker. In addition, the subjects behaved according to their roles in the presented scenarios. On a scale where 1 indicates role-inconsistent behavior and 5 indicates role-consistent behavior, the test participants answered on average with *M* = 4.2 (*SD* = 0.5), which shows that the participants knew their role characteristics. The majority of the participants (61.81%) identified well or very well with the role of a firefighter. A total of 21.82% said that they could identify with the role of a firefighter fairly well, 13.64% related poorly to the role, and 2.73% could not identify with the role. A total of 51.66% of the participants could identify with the role of a senior firefighter well or very well. A total of 21.67% of the participants stated that they could identify with the role as a senior firefighter fairly well, 18.33% related poorly, and 8.33% did not relate at all. Junior and senior firefighters experienced the same degree of threat in the non-CAS, *t*(118) = 1.10, *p* = 0.272, and in the CAS, *t*(118) = 1.21, *p* = 0.229. Therefore, in the following calculations, no distinction is made for the role factor.

#### Manipulation check

The perception of situation characteristics differed significantly between action phase 1 (non-CAS) and action phase 2 (CAS), *t*(38) = −13.33, *p* < 0.001. In action phase 1, participants were more likely to perceive characteristics of a non-CAS (*M* = 2.4, *SD* = 0.8), while in action phase 2, they were more likely to perceive characteristics of a CAS (*M* = 4.6, *SD* = 0.7). The perception of the manipulation of feedback *via* situation specificity differed significantly between G1 and G2, t(78) = 8.22, p < 0.001. Participants who received situation-unspecific feedback evaluated the feedback as more inadequate in relation to the mastered mission (*M* = 3.5, *SD* = 1.0) compared to participants who received situation-specific feedback (*M* = 2.0, *SD* = 0.6). Therefore, both manipulations had worked.

#### Structure testing of triggers and needs

The confirmatory factor analysis (CFA) revealed that triggers and needs are distinct constructs. As expected, Model 2, with two distinct latent factors, provided a better fit than Model 1, with only one g-factor. The model estimation did not terminate normally. Parameter estimates for one item of the trigger relationship-related disrespect had negative variances. We followed the instructions of Urban and Mayerl (2014) and removed outliers from the analysis. The model fit for both models was acceptable, although the goodness-of-fit statistic was statistically significant for each model. An overview of the goodness-of-fit indices of both models is presented in Table 2. The chi^2^-difference test showed that the hypothesized Model 2 represented the data significantly better than Model 1 (Δχ^2^[1] = 4.26, *p* < 0.01).

**Table 2:** Goodness-of-fit indices of alternative CFA models.

Model	χ^2^	*df*	CFI	TLI	RMSEA	SRMR	AIC	BIC
1. g-factor	385.112	244	0.871	0.855	0.073	0.109	6,543	6,699
2. First-order correlated factor	370.056	243	0.901	0.888	0.066	0.118	6,522	6,681

χ^2^, chi-square for all models is *p* < 0.01; df, degrees of freedom; CFI, comparative fit index; TLI, Tucker–Lewis index; RMSEA, root-mean-square error of approximation; SRMR, standardized root-mean-square residual; AIC, Akaike information criterion; BIC, Bayesian information criterion.

### Hypotheses-related results

#### Hypothesis 1a

Non-CAS (action phase 1) and CAS (action phase 2) activated different triggers, *F*(4,36) = 39.51, *p* < 0.001, η_p_^2^ = 0.81. Non-CAS and CAS differed in trigger activation for experience of failure, *F*(1) = 148.88, *p* < 0.001, η_p_^2^ = 0.79, and hindered action regulation, *F*(1) = 57.66, *p* < 0.001, η_p_^2^ = 0.60, for the no feedback group (G3). In the CAS (action phase 2), participants who did not receive feedback (G3) perceived a stronger activation of experience of failure (*M* = 4.6, *SD* = 0.9) and hindered action regulation (*M* = 3.1, *SD* = 1.1) triggers than in the non-CAS (action phase 1; experience of failure [*M* = 2.4, *SD* = 1.2] and hindered action regulation [*M* = 1.6, *SD* = 0.9]). Thus, Hypothesis 2a was supported. The CAS activated a stronger experience of failure and hindered action regulation as insufficiency triggers compared to the non-CAS (Table 1).

#### Hypothesis 1b

After the CAS (action phase 2), participants differed in trigger activation depending on their feedback condition (negative, situation-specific feedback; negative, situation-unspecific feedback; or no feedback), *F*(4,113) = 945.53, *p* < 0.001, η_p_^2^ = 0.97. *Post hoc* tests showed that G1 (*M* = 2.7, *SD* = 1.1) and G2 (*M* = 2.4, *SD* = 0.8) differed significantly from G3 (*M* = 1.8, *SD* = 0.6) in the activation of the performance-related disrespect trigger. There was no significant difference between G1 and G2 in the activation of performance-related disrespect. Additionally, participants receiving different feedback did not differ in activation of the relationship-related disrespect trigger, G1 (*M* = 1.2, *SD* = 0.4), G2 (*M* = 1.1, *SD* = 0.4), G3 (*M* = 1.0, *SD* = 0.2). Therefore, Hypothesis 2b was only partially supported. After CAS, negative feedback that was situation-nonspecific or situation-specific activated the performance-related disrespect trigger compared to no feedback. Contrary to our hypothesis, we found no effect of the feedback’s situation specificity on performance-related trigger activation. Furthermore, none of the feedback conditions (G1-3) had an effect on relationship-related trigger activation.

#### Hypothesis 2a

**Figure 3:**
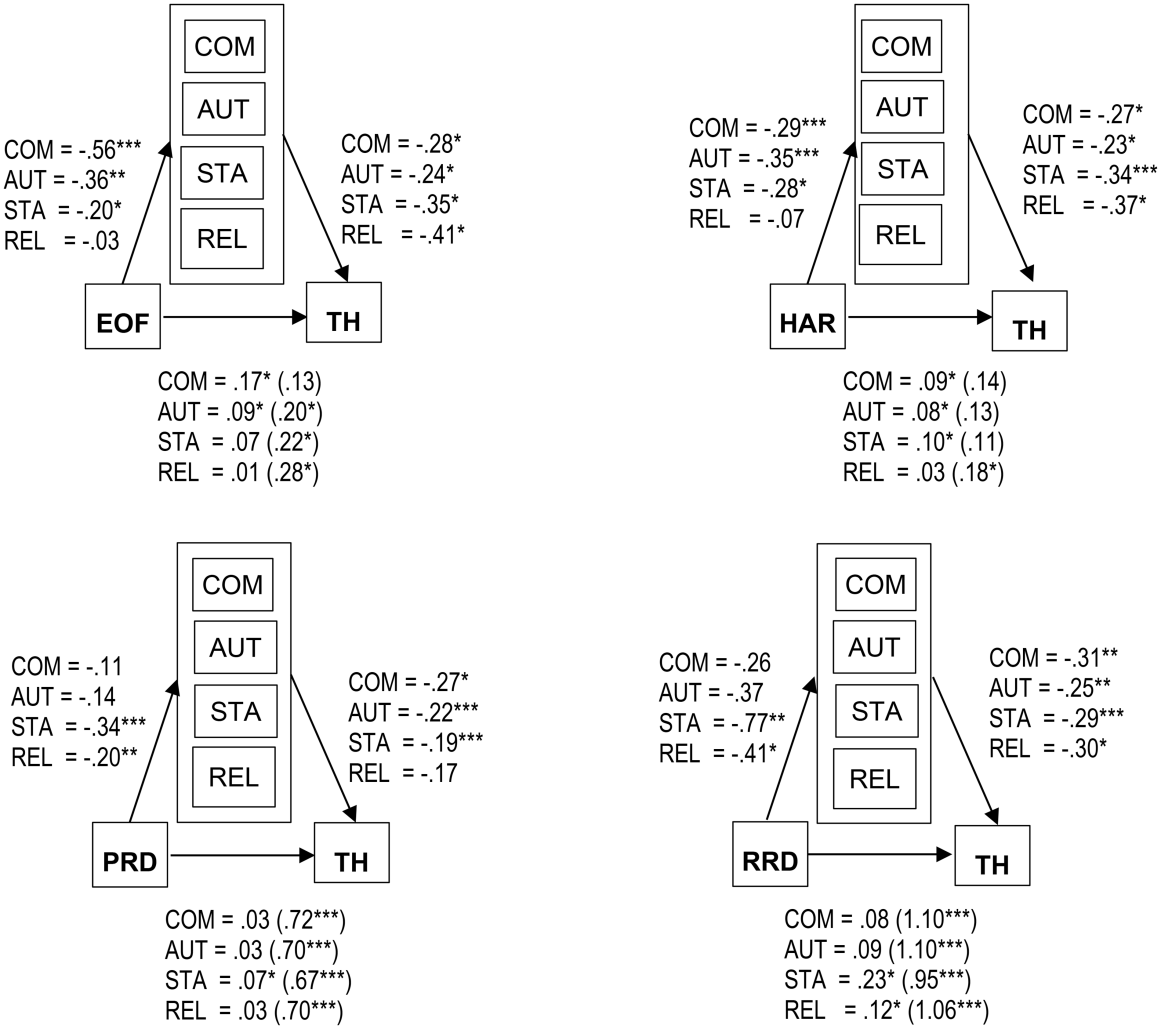
Indirect and direct effects in parentheses from mediation analyses for the trigger-needs-threat relationships. TH, threat; EOF, experience of failure; HAR, hindered action regulation; PRD, performance-related disrespect; RRD, relationship-related disrespect; COM, need for competence; AUT, need for autonomy; STA, need for status; REL, need for relatedness. ^*^p < 0.05; ^**^p < 0.01; ^***^p < 0.001.

We found a significant direct effect of experience of failure on threat (*r* = 0.28, *p* < 0.003) and significant indirect effects of experience of failure on threat with the need for competence (*b* = 0.156, 95% BCa CI[0.0076, 0.3231]) and need for autonomy (*b* = 0.086, 95% BCa CI[0.0196, 0.1860]) as mediators (see Figure 3). Furthermore, we did not find a direct effect of hindered action regulation on threat (*r* = 0.14, *p* = 0.082). However, we found a significant indirect effect of hindered action regulation on threat with the need for competence (*b* = 0.079, 95% BCa CI[0.0177, 0.1596]), need for autonomy (*b* = 0.079, 95% BCa CI[0.0181, 0.1498]), and need for status (*b* = 0.095, 95% BCa CI[0.0361, 0.1748]) as mediators (see Figure 3). As hypothesized, the results indicated that the relationship between the experience of failure and hindered action regulation (as insufficiency triggers) and threat was mediated by the needs for competence and autonomy. Contrary to hypotheses 2a regarding the relationship between hindered action regulation and threat, we found a mediating effect of the need for status.

#### Hypothesis 2b

We found a significant direct effect of performance-related disrespect on threat (*r* = 0.70, *p* < 0.001). Moreover, we found a significant indirect effect of performance-related disrespect on threat with the need for status (*b* = 0.066, 95% BCa CI[0.0089, 0.1360]) as a mediator (see Figure 3). Furthermore, we found a direct effect of relationship-related disrespect on threat (*r* = 0.96, *p* < 0.001). There were indirect effects of relationship-related disrespect on threat with the need for status (*b* = 0.227, 95% BCa CI[0.0576, 0.4719]) and the need for relatedness (*b* = 0.123, 95% BCa CI[0.0018, 0.3295]) as mediators (see Figure 3). With regard to the relationship-related disrespect trigger, the results support Hypothesis 2b, such that the relationship of relationship-related disrespect and threat was mediated by the needs for relatedness and status. In the case of performance-related disrespect, we found a mediating effect of the need for status only.

## Discussion

In an experimental setting, the activation of threat triggers in action and postaction phases of CAS and the mediating role of needs. First, triggers were activated due to situational characteristics in the action phases (CAS vs. non-CAS) and due to feedback in the postaction phases (situation-specific vs. situations-nonspecific vs. no feedback). More specifically, our results indicate that situational characteristics of the action phase of a CAS (compared to non-CAS) activated both insufficiency triggers: the experience of failure and hindered action regulation (Hypothesis 1a). In the postaction phases, negative situation-specific and situation-nonspecific feedback activated the trigger of performance-related disrespect but not that of relationship-related disrespect (Hypothesis 1b). The temporal perspective of teamwork as well as the differentiation of the two forms of disrespect and the two forms of insufficiency highlight the potential prevalence of threat triggers in a CAS. Second, we strengthened the evidence of the crucial role of needs in the threat mechanism. Specific triggers frustrated specific needs and led to threats (Hypotheses 2a and 2b).

Our results indicate that individuals in the action phases of a CAS perceive insufficiency in two ways: experiences of failure and hindered action regulation. An experience of failure due to an internal attribution of insufficient skills and abilities is postulated as a threat trigger in the SOS theory (Semmer et al., 2007, 2019). In the context of a CAS, we identified hindered action regulation (describing the external attribution of insufficiency to environmental or contextual factors) as another trigger. The fact that events perceived as hindrances for goal attainment are stressful is also postulated in challenge hindrance models of work stress (CHMs, Cavannaugh et al., 2000). Even though hindrances are seen as a separate kind of stress experience in CHMs, some empirical studies suggest that triggers such as bureaucratic constraints, which are otherwise associated with hindrances, can also trigger threats (Brady and Cunningham, 2019). This is in line with our findings showing that perceptions of insufficiency in the action phases of a CAS can be attributed not only to internal but also to external factors. This highlights the multifaceted risk for activating threat triggers and underscores the danger of threats.

Additionally, the results of this study showed that there is threat potential in feedback in postaction phases. Even though postaction feedback and reflection processes are important for the learning and adaptivity of teams (Konradt et al., 2015), several studies indicate that negative feedback is accompanied by threats (e.g., Burnette et al., 2010; Dunn and Dahl, 2012; Lee-Won et al., 2017). There is a great deal of research offering advice related to giving feedback. For example, Gabelica et al. (2012) postulate in their review that feedback is effective if it is accurate, given on time, and is nonthreatening. What exactly is experienced as threatening is not specified more precisely. Based on our study results, we did not find any evidence that situation specificity in feedback was decisive for perceptions of disrespect and, in turn, for threats. Our study results showed that negative feedback, whether situation-specific or not, signals performance-related disrespect. The negative feedback emphasizes the failure that participants have previously experienced in the CAS itself (action-related experience of failure and hindered action regulation as threat triggers). Although participants made an effort, their performance was rated poorly, indicating a kind of disrespect (*cf.* Grover, 2013). Due to the strength of the negative feedback (“You failed…”), participants possibly did not perceive subsequent attributions to the situational characteristics. Furthermore, it is not surprising that any feedback (situation-specific feedback vs. situation-nonspecific feedback vs. no feedback) activated relationship-related disrespect as a threat trigger. In all feedback conditions, feedback was given in a friendly tone according to the standards of interpersonal interactions and the consideration of human dignity. Due to ethical limitations, relationship-related disrespect could not be manipulated and decisively analyzed in this experiment. Nevertheless, our findings implied that even in simple feedback processes, there is a high risk of the perception of disrespect. This shows the great risk of activating threat triggers in postaction phases, which also underscores the danger of threats.

Moreover, this study confirmed that the way individuals perceive, evaluate, and respond to triggers is determined by needs, so that needs are responsible for whether events act as triggers and lead to threats. Therefore, the danger of threats lies not only in the experience of insufficiency (i.e., the experience of failure and hindered action regulation) and disrespect (i.e., relationship-and performance-related disrespect), but it also depends on needs. Needs are more than goals and are essential for optimal functioning and growth (Manganelli et al., 2018). Needs guide a variety of basic human processes, including how individuals define themselves in the sense of who they are and who they want to be (Ashforth and Schinoff, 2016). Therefore, triggers that frustrate needs target the individual in core ways, which also strengthens the danger of threats.

### Theoretical and practical implications

First, we confirmed the triggers identified in the SOS theory within the context of a CAS, and we extended them with the perspective of action and postaction phases of teamwork (Marks et al., 2001). This highlights that the danger of threats exists not only in the situation itself but also in the aftermath. Nevertheless, we showed that in the context of a CAS, triggers can manifest in different forms; for instance, disrespect was separated into relationship-and performance-related disrespect. This subdivision of triggers can help individuals to be sensitive to potential threat-triggering events. Becoming aware of triggers may be an important aspect of avoiding threats. For example, if supervisors know that their performance feedback has a threatening effect by signaling disrespect, they can interact more attentively with their staff, such as by taking needs into account (van Quaquebeke and Felps, 2018). Second, we strengthened the assumption of the needs-based mechanism as an explanatory approach to threats postulated in the framework of the SOS theory (Semmer et al., 2007). Needs are well known as an important factor influencing appraisal processes in motivational research (e.g., Deci et al., 2017) or in stress research in general (e.g., Rohmert, 1983). In this study, we showed that needs are specific for threat appraisals. Therefore, we render threats as a stress experience potentially more explainable and predictable.

Overall, our study highlighted that the action and postaction phases of CAS pose a variety of risks for experiencing threats within and after an emergency scenario. Research on teams in CAS scenarios provides numerous recommendations on how teams can master a CAS successfully or how teams can remain able to act during a CAS (e.g., Maynard et al., 2018). For example, in complex crises and under time pressure, teams need a highly disciplined communication structure and a common understanding of the situation for a rapid response (Uitdewilligen and Waller, 2018). To help individuals cope with these risks, needs are important mediators. Need frustration explains why threat experiences occur. Consequently, reattribution and reflection may be an approach to reduce the frustration of needs and the occurrence of threats in the case of activated triggers.

### Limitations

Despite the promising results of this study, some critical remarks must be made. First, there are some factors to mention that possibly influenced the study results. For example, participants were able to register as a team (73.33% of the participants were friends). It is known from team research that especially communication and coordination processes are influenced by psychological safety or trustworthiness, as it is common among friends (e.g., Edmondson, 1999). Furthermore, the extent to which participants identified with their role and the team might also have affected the study results. Several studies indicate that a strong identification with the team affects performance and motivation (e.g., Solansky, 2011). However, in our study, all participants indicated that at least less than 9% of the senior firefighters and less than 3% did not identify with their role. Subjects in our study also seem to be able to identify with the team. On average, they respond to statements about team identification that they “tend to agree.

Second, the computer-based simulation of CAS, as well as the sample of students who assumed the role of a firefighter, may lack ecological validity. However, initial findings from interviews with paramedics and police officers confirm the relevance of the triggers and needs for experiencing threats in the field (Rynek and Ellwart, 2020). Correlative data in a sample of leaders working part-time also confirm the mediating effect of needs in the field (Rynek et al., 2021). Additionally, the experimental design in this study allows for a causal interpretation of the effects of the triggers and needs in and after CAS.

Third, even though research indicates that there are often multiple triggers and needs involved in one threat situation (e.g., Anshel, 2000), this study provides as little evidence of the effects of multiple triggers and needs within a single situation as it does of the effects of specific trigger-need combinations on threat experiences. In the real world, we have many processes and effects happening at the same time. In a survey study, Rynek et al. (2021) provided evidence that needs mediate the relationship between triggers and negative outcomes in a study focusing on dysfunctional support (i.e., social support that is accompanied by reproaches) as a threat trigger for part-time leaders and their need for relatedness. However, in our experimental study, simultaneously, multiple triggers and needs were operationalized in the laboratory setting. We manipulated individual threatening events in an experimental setting and tested both the perception of these events as triggers and the extent to which they frustrated needs and thus led to threats. It is important to understand the experiment-related causal effects before interaction effects become the focus of research.

Fourth, although we assigned different role descriptions (senior vs. junior firefighter) to the team members, we did not find any differences between threat experiences among team members’ roles (see Preliminary Results). Individuals take on different roles depending on the context (e.g., roles as mother, friend, supervisor, colleague; Sluss et al., 2011). The strength of a need depends on predispositions but also contextual features (Ashforth and Schinoff, 2016). By assigning responsibility to one team member and thereby making senior firefighters aware of being the decision-making person in the team, we created a context in which status beliefs become salient. However, we did not find any differences between the roles of senior and junior firefighters in their threat experiences. Although the senior firefighter had an important position in the team due to being responsible for the actions and performance of the team, the assignment of responsibility might have stimulated reflection on the standing within the team for the junior, as well. Based on our role manipulation, it was not possible to make claims about role-related differences in needs and thus threat experiences. The general understanding of the trigger-need mechanism was revealed for the junior and senior roles. In future studies, the manipulation of role differences should be improved.

### Future perspectives

We have shown that handling CAS and the feedback that follows may activate triggers and result in the perception of threats. CAS are often handled in a team setting that might also influence the experiences of threat. The team perspective provides triggers, such as disrespectful feedback from colleagues or supervisors (Anshel, 2000) but also offers approaches to compensate for frustrated needs (e.g., social support; Bakker and Demerouti, 2007); thus, teamwork can strengthen or hinder the potential for threats. The systematic input-process-output models (Mathieu et al., 2008) indicate concepts to avoid triggers (e.g., psychological safety) and concepts that strengthen individual needs as a buffer (e.g., team cohesion), which should be a topic in future research.

Furthermore, especially in the context of CAS, there can often be multiple triggers and needs involved in one threat situation. For example, while a firefighter’s action is hindered by too many injured people and a lack of resources to save them, the firefighter tries to protect his own life and limb against the fire (Jacobsson et al., 2015). Impaired regulation of action and physical danger act as triggers. A frustrated need for physical integrity, which should inevitably be activated when experiencing physical danger, is, according to Maslow (1943), a need that is more significant than others. Therefore, it is conceivable that simply the activation of the physical danger trigger has stronger effects than others and that the combination of many triggers increases threat experiences. Therefore, the interaction of triggers and needs should be the subject of future research.

Finally, future research should consider the investigation of threats from different types of professionals, such as firefighters and police officers, into account. Unlike a student participant who learns the firefighter role in the short term, professional firefighters’ role expectations are consolidated. In future research, instead of students receiving feedback from the principal investigator, real firefighters can run the Networked-fire-chief-simulations in the laboratory setting and receive feedback from their supervisors.

## Conclusion

This study emphasizes the potential danger of threats in CAS not only in action but also postaction phase of team reflection and feedback. Knowledge about the different triggers and effects of emergency workers’ needs may offer the chance to find new approaches to understand, avoid, or cope with threats in CAS. Through the differentiation of action and postaction phases, we underline the diversity of triggers and also may suggest possible resources for mastering CAS successfully.

## Data availability statement

The raw data supporting the conclusions of this article will be made available by the authors upon request.

## Ethics statement

The studies involving human participants were reviewed and approved by Ethical Committee, University of Trier, Universitätsring 15, 54286 Trier. The patients/participants provided their written informed consent to participate in this study.

## Author contributions

MR and TE contributed to conception and design of the study. MR organized the database, performed the statistical analysis, and wrote the first draft of the manuscript. TE wrote sections of the manuscript. All authors contributed to the article and approved the submitted version.

## Conflict of interest

The authors declare that the research was conducted in the absence of any commercial or financial relationships that could be construed as a potential conflict of interest.

## Publisher’s note

All claims expressed in this article are solely those of the authors and do not necessarily represent those of their affiliated organizations, or those of the publisher, the editors and the reviewers. Any product that may be evaluated in this article, or claim that may be made by its manufacturer, is not guaranteed or endorsed by the publisher.
